# Temporal coupling between papillary muscle strain and left ventricular untwisting during diastole

**DOI:** 10.1093/ehjimp/qyag117

**Published:** 2026-06-24

**Authors:** Nidhal Bouchahda, Fabian Scheipl, Haithem Hbid, Mohamed Yessine Kallela, Meriem Hentati, Mejdi Ben Messaoud, Mezri Maatouk, Aymen Najjar

**Affiliations:** Cardiology A Department, Fattouma Bourguiba University Hospital, Monastir University, Rue du 1er juin 1955, Monastir 5000, Tunisia; Munich Center for Machine Learning, Ludwig-Maximilians-Universitat Munchen, Munich, Germany; Cardiology A Department, Fattouma Bourguiba University Hospital, Monastir University, Rue du 1er juin 1955, Monastir 5000, Tunisia; Cardiology A Department, Fattouma Bourguiba University Hospital, Monastir University, Rue du 1er juin 1955, Monastir 5000, Tunisia; Cardiology A Department, Fattouma Bourguiba University Hospital, Monastir University, Rue du 1er juin 1955, Monastir 5000, Tunisia; Cardiology A Department, Fattouma Bourguiba University Hospital, Monastir University, Rue du 1er juin 1955, Monastir 5000, Tunisia; Radiology Department, Faculty of Medicine, University of Monastir, Monastir, Tunisia; Cardiology A Department, Fattouma Bourguiba University Hospital, Monastir University, Rue du 1er juin 1955, Monastir 5000, Tunisia

**Keywords:** left ventricular untwisting, papillary muscle strain, cardiac torsion, diastolic function, functional data analysis

## Abstract

**Aims:**

Left ventricular (LV) untwisting is classically considered a passive recoil phenomenon. However, during conditions of shortened diastole, passive mechanisms alone may be insufficient to ensure effective diastolic suction. We hypothesized that both anterolateral papillary muscle (APM) and posteromedial papillary muscle (PPM) contribute actively, alongside passive elastic recoil, to LV untwisting during diastole.

**Methods and results:**

In this prospective study, individuals with normal transthoracic echocardiograms underwent two-dimensional speckle-tracking imaging. Longitudinal strain of APM and PPM was obtained, alongside LV torsion measurements. Functional data analysis and penalized function-on-function regression were used to model the temporal association between papillary muscle strain and LV untwisting. A total of 75 patients were enrolled in the study, with a mean age of 37.2 ± 14.9 years. Of these, 16 (21.3%) were female. Hypertension was present in 12 (16%) patients while 7 (9.3%) patients had diabetes. The mean peak torsion measured 25 ± 10 degrees. Maximum contractions of APM and PPM were −17 ± 3.8% and −17 ± 3.7%, respectively. The mean peak LV torsion occurred at 41% of the cardiac cycle and peak global longitudinal strain at 43%, while peak APM and PPM contractions occurred significantly later, at 51% (*P* < 0.001). Functional regression revealed a significant, time-dependent relationship between papillary muscle contraction and untwisting. Greater APM and PPM contraction was associated with enhanced LV untwisting, during early and mid-diastole.

**Conclusion:**

Papillary muscles actively contract during diastole and are associated with LV untwisting in a temporally coordinated manner.

## Graphical Abstract

**Graphical Abstract:**
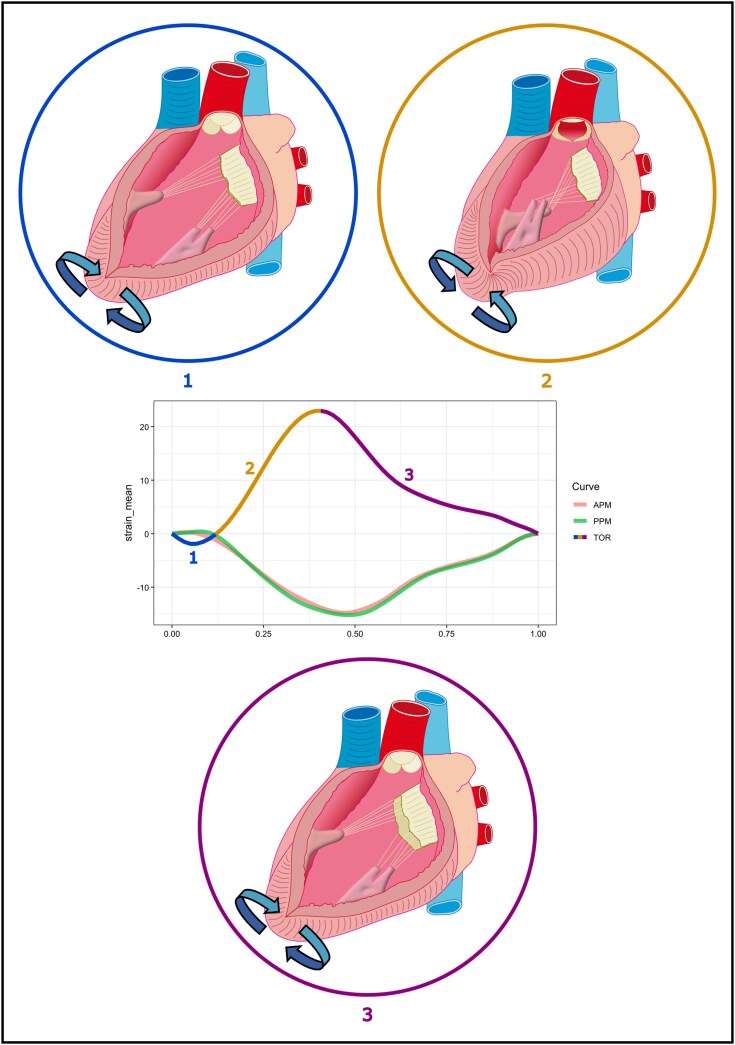

## Introduction

The myocardial twist is a fundamental component of left ventricular (LV) function, significantly influencing its mechanical performance. This twisting motion arises from the unique arrangement of helically oriented myocardial fibres, which induce opposing rotational movements at the base and apex of the LV during systole.^1–3^ The resulting torsional deformation has classically been proposed to store mechanical energy within the ventricular myocardium, which is then released during diastole. This release is thought to contribute to ventricular untwisting, thereby facilitating ventricular suction and early diastolic filling.^4^ Accordingly, LV untwisting has long been described as a predominantly passive phenomenon driven by elastic recoil of the myocardial fibres. However, several features of untwisting, including its rapid onset, precise temporal coupling with isovolumic relaxation, and spatial coordination, are difficult to reconcile with a purely passive mechanism.^5–7^ Taken together, these observations support the presence of an active component that contributes to, or regulates, LV untwisting in parallel with elastic recoil. The papillary muscles (PMs), small muscular projections linking the basal and apical regions of the LV, have been shown in animal studies to reach peak contraction during diastole, coinciding with the untwisting phase.^8–12^ This unique anatomical configuration combined with timing of their contraction led us to hypothesize that PMs may play an active role, alongside passive recoil, in the regulation of LV untwisting. This hypothesis is further supported by observations from this bench model (see Supplementary data online, *Video S1*). Accordingly, the objectives of this study are as follows: (i) to determine whether PM contraction peaks during diastole in humans and (ii) to assess the potential association of PMs to LV untwisting.

This study is observational. While an observational design cannot formally establish causal mediation, the timing of PM contraction and the anatomical configuration of the PMs together support the hypothesis of an active PM contribution to diastolic mechanics.

## Methods

### Patient population

This prospective study included patients who underwent comprehensive transthoracic echocardiographic evaluation. Participants in sinus rhythm, with normal echocardiographic findings, were included. Non-inclusion criteria included history or suspicion of coronary artery disease, heart failure, atrial fibrillation, history of chemotherapy, uncontrolled hypertension, or diabetes. Exclusion criteria were primarily LV hypertrophy and non-feasibility of PM longitudinal strain assessment. PM strain assessment was feasible in 75 of the screened patients; the remainder were excluded for poor PM tracking quality.

The study protocol was approved by the Ethics Committee of Fattouma Bourguiba Hospital, and all participants provided written informed consent.

### Echocardiographic parameters

Transthoracic echocardiographic images were obtained using a VIVID E9 ultrasound system (GE Healthcare, Waukesha, WI, USA) equipped with an M5 probe. All recordings were digitally stored and analysed offline using EchoPAC software version V113 (GE Healthcare).

### PM longitudinal strain imaging

The anterolateral papillary muscle (APM) was visualized in a modified, anteriorly tilted apical four-chamber view (see Supplementary data online, *Video S2*), while the posteromedial papillary muscle (PPM) was imaged in a modified apical long-axis three-chamber view (see Supplementary data online, *Video S3*). Each PM was tracked from its base of attachment to its tip, excluding any chordae tendineae. Care was taken to have the PM visualized throughout the entire cardiac cycle (see Supplementary data online, *Videos S4* and *S5*). Visual confirmation of accurate tracking throughout the entire cardiac cycle was required before strain data were extracted.^13,14^ Two to three cardiac cycles were recorded per patient for analysis.

### LV torsion by two-dimensional speckle-tracking

Short-axis images at the basal level were acquired at the mitral valve plane. For apical images, the probe was initially placed in a standard apical four-chamber position and then angled downward to obtain a circular cross-sectional view of the LV apex, minimizing visualization of the right ventricle. Special attention was given to acquiring truly circular short-axis views. Regions of interest were manually adjusted to optimize tracking quality, and echogenic pericardium was excluded. Rotational deformation values were derived after applying circular strain analysis to both apical and basal short-axis views. LV twist was then calculated as the difference between apical and basal rotation across the cardiac cycle.^1,15,16^

### Strain values

**Figure 1:**
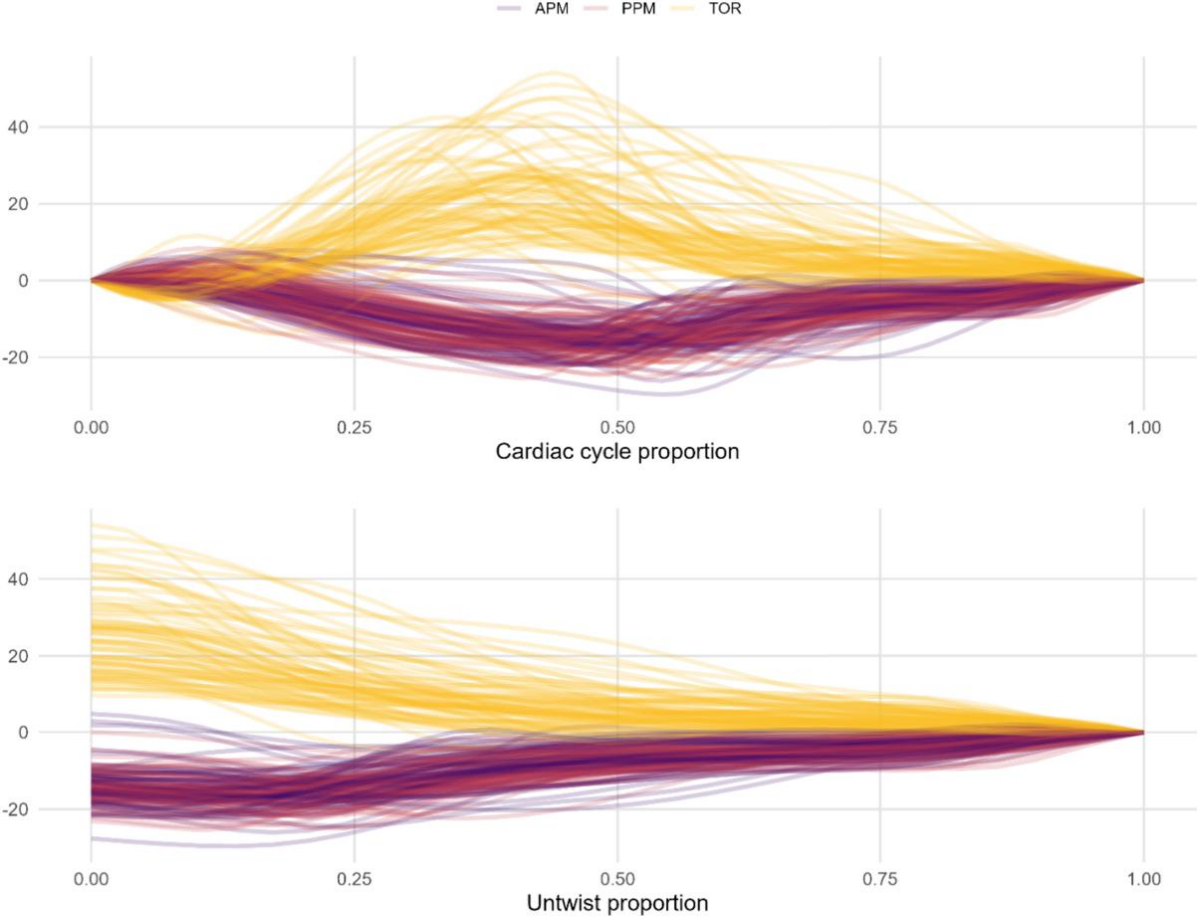
Time-normalized papillary muscle strain and left ventricular torsional strain curves over the entire cardiac cycle (top) and during the untwisting phase only (bottom). APM, anterior papillary muscle; PPM, posterior papillary muscle; Tor, torsion.

**Figure 2:**
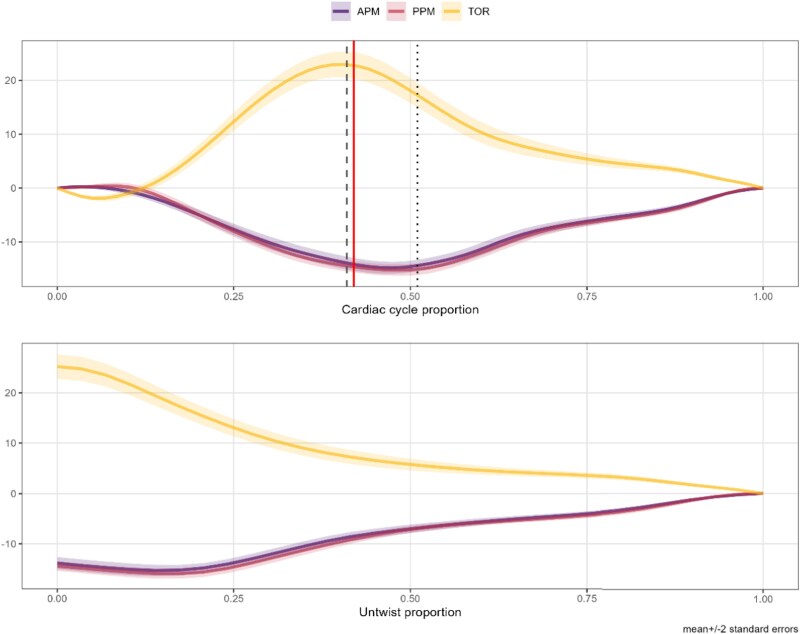
Mean strain curves for left ventricular torsion and the anterior and posterior papillary muscles over the entire cardiac cycle (top) and during the untwisting phase (bottom). In the lower panel, time is re-referenced to the instant of peak LV torsion identified in the upper panel. In the top panel, the blue dashed vertical line indicates the mean timing of peak LV torsion, the black dashed vertical line denotes the mean timing of peak anterior and posterior papillary muscle contraction, and the red vertical line marks aortic valve closure. APM, anterior papillary muscle strain (%); PPM, posterior papillary muscle strain (%); TOR, left ventricular torsion (degrees). Light ribbons represent pointwise ±2 standard SE.

Strain and torsion curves were exported as numerical time series using the *Store Trace* function of EchoPAC.^17^ Frame rates ranged from 50 to 70 frames per second, and zero strain was defined at LV end-diastole. Throughout the manuscript, we distinguish two coordinate systems. Cardiac-cycle proportion refers to the full R-R interval rescaled to [0, 1] (60 samples), used for whole-cycle comparisons and for the timing of peak strain and peak torsion. Untwisting-phase proportion is a within-patient relative coordinate that rescales the patient-specific interval from peak LV torsion to end-of-cycle to [0, 1] (30 samples); this aligns the diastolic interval across patients despite differing heart rates and is used for the function-on-function regression (*Figures 1* and *2*).^18^

Unlike prior studies that summarize untwisting by the nadir of the untwisting rate, a single derived value, we analysed untwisting as a continuous mechanical process from peak twist to end-diastole. While the untwisting rate nadir is mathematically convenient, it lacks intuitive mechanical meaning and is difficult to conceptualize in terms of myocardial motion, making mental visualization of the underlying process challenging. More importantly, reducing untwisting to a single point obscures early diastolic mechanics, particularly the onset of untwisting. Our approach preserves the temporal evolution of untwisting and enables assessment of its relationship with PM deformation across the entire untwisting interval, with specific emphasis on its initiation.

### Statistics

Categorical data were presented as absolute numbers and percentages, while continuous variables were summarized using the mean and standard deviation. To include the strain curve as a covariate, we applied functional data analysis.^19^ The objective was to predict LV untwist using PM strain curve as a functional predictor. We assumed that LV torsion at a given time point (s) depends exclusively on PM contraction occurring at that same moment (t = s). Therefore, we adopted a concurrent function-on-function regression approach to model this temporal relationship,^20^ i.e. the effect of PM strain at time t on torsion is evaluated at the same time t. This choice was made (i) for identifiability at *n* = 75 functional observations, since a general historical/lagged surface would involve substantially more degrees of freedom; (ii) for physiological plausibility on the millisecond time scale of intracardiac mechanical coupling; and (iii) for interpretability. Lagged, cumulative, and APM × PPM interaction effects were not modelled and are discussed in Limitations as natural extensions for larger cohorts. Analysis was limited to untwisting phase of the strain curves starting at the peak of torsion. Alongside the PM strain curves, age, gender, and heart rate were included as scalar covariates. One observation had missing values for heart rate, which was imputed using the mean. Data preparation and curve/result visualizations were performed using the tf and mgcViz packages.^21,22^ The function-on-function regression analysis was carried out using penalized function on function regression from the refund package.^23^ The function-on-function regression produces a coefficient surface rather than a single number. At each combination of (PM strain, untwisting-phase time), the surface answers a single question: ‘If two otherwise comparable patients differ in their PM strain at this point of diastole, by how much do we expect their simultaneous LV untwist to differ?’ PM strain is mean-centred at each time point, so negative horizontal values correspond to stronger-than-average contraction and positive values to weaker-than-average contraction. The colour encodes the slope (effect on torsion, in degrees) of that local association: red = stronger PM contraction is associated with greater simultaneous untwisting; blue = the opposite; white = no statistically significant association at alpha = 0.05; dark grey = no observed data in that strain-time region. Smoothing across both axes implements the assumption that biologically adjacent strain-time regions share similar effects; smoothing parameters are selected by REML.

R software was used to perform all the above analysis.

## Results

A total of 75 individuals were enrolled in the study, with a mean age of 37.2 ± 14.9 years. Of these, 16 (21.3%) were female. Hypertension was present in 12(16%) patients while 7 (9.3%) patients had diabetes. The average heart rate was 75 ± 13.5 beats per minute (*Table 1*).

**Table 1:** Baseline characteristics of the study population

	Population (*n* = 75)
Age, mean (SD), years	37.4 (14.9)
Female, *n* (%)	16 (21.3%)
Hypertension, *n* (%)	12 (16.0%)
Diabetes, *n* (%)	7 (9.3%)
Weight, mean (SD), kg	75.0 (13.3)
Height, mean (SD), cm	173 (8.21)
Heart rate, mean (SD), bpm	75.3 (13.7)
Torsion peak, mean (SD), degrees	25.2 (10.6)
Torsion peak time, mean (SD)	0.41 (0.06)
GLS peak, mean (SD), %	−17.3 (2.20)
GLS peak time, mean (SD), %	0.43 (0.05)
APM peak, mean (SD), %	−17.1 (3.80)
APM peak time, mean (SD), %	0.509 (0.0765)
PPM peak, mean(SD), %	−17.8 (3.73)
PPM peak time, mean(SD), %	0.509 (0.0791)

All strain curves were averaged over two to three cardiac cycles. The mean peak torsion measured 25 ± 10 degrees. Maximum contractions of APM and PPM were −17 ± 3.8% and −17 ± 3.7%, respectively. *Figure 2* illustrates the mean LV torsion curve alongside average APM and PPM strain curves across the cardiac cycle. Peak LV torsion occurred at 0.41 ± 0.06 of the cardiac cycle proportion, whereas peak APM and PPM contractions occurred later, both at 0.51 ± 0.08. Peak global longitudinal strain (GLS) was reached at 0.43 ± 0.05. The timing differences were statistically significant, with peak PM contraction occurring later than peak torsion [*P* < 0.001; 95% confidence interval (CI) for difference: 0.08–0.11] and later than GLS peak timing (*P* < 0.001; 95% CI for difference: 0.06–0.09).

Functional regression analysis revealed significant, time- and magnitude-dependent associations between the contractions of both APM and PPM and LV untwisting.

In this functional regression model, the relationship between PMs strain and LV untwisting was represented by a regression coefficient surface rather than a single line, as seen in typical conventional models. This surface illustrates how the effect of PM contraction on untwisting varies across both the magnitude of PM strain (X-axis) and time during untwist (Y-axis).

Strain values were mean-centred at each time point; therefore, negative X-values represent PM strain below the average at that time, while positive X-values represent PM strain above the average.

The coefficient surface is visualized using a colour map to indicate the direction and statistical significance of the association:

- Red denotes a significant positive association
- Blue denotes a significant negative association
- White indicates no significant association (i.e. the 95% CI included zero)
- Dark grey indicates regions lacking patient data for the corresponding strain-time combinations, preventing model estimation

**Figure 3:**
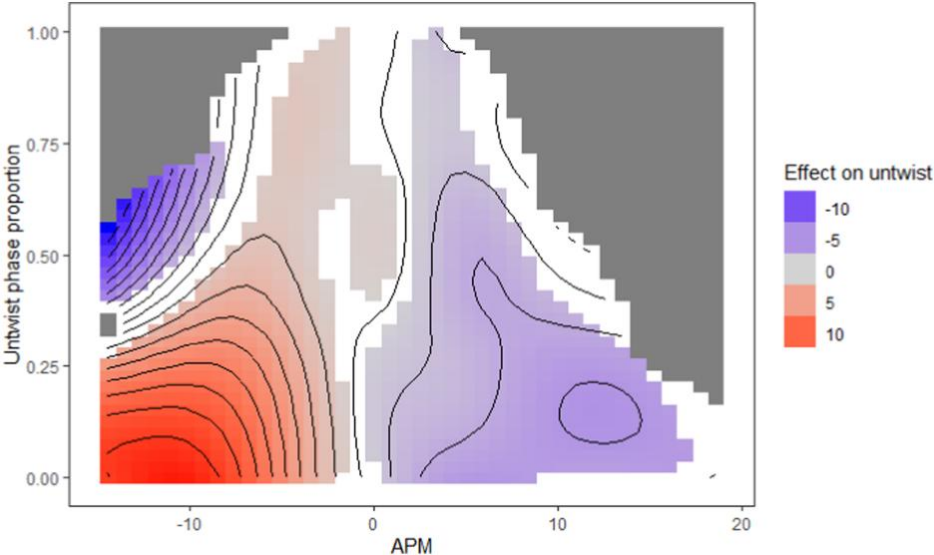
Effect of anterior papillary muscle on untwisting. Y-axis: Proportion of untwist phase X-axis: Deviation from mean APM strain values (%). Positive values indicate above-average strain, while negative values represent below-average strain. Colour gradients reflect the direction and strength of the association with left ventricular untwisting. White areas denote regions where the regression coefficients are not significantly different from zero. Dark grey areas indicate regions without observed data. APM, anterior papillary muscle. Example interpretation: An APM strain value of −10% (i.e. 10 units below the mean, indicating stronger contraction) is associated with a strong significant positive effect on untwisting (intense red colour) at ∼0.1 of the untwist phase.

We found that stronger APM contraction (i.e. more negative strain values, below the mean) was positively associated with LV untwisting during early diastole, whereas weaker contraction (positive values, above the mean) showed a negative association. The association strength increased at more negative strain values, indicating that greater APM shortening was linked to more pronounced untwisting. This effect was primarily observed during the transition from peak twist to untwist. In later diastole, however, extremely negative APM values became negatively correlated with untwisting, suggesting a change in the functional relationship between APM contraction and LV relaxation (*Figure 3*).

**Figure 4:**
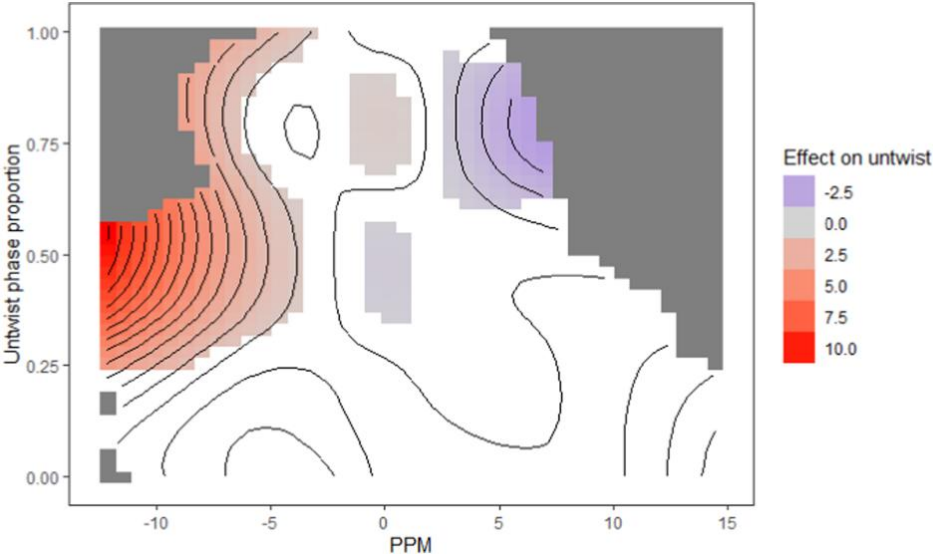
Effect of posterior papillary muscle on untwisting. Y-axis: Proportion of untwist phase; X-axis: Deviation from mean PPM strain values (%). Positive values indicate above-average strain, while negative values represent below-average strain. Colour gradients reflect the direction and strength of the association with left ventricular untwisting. White areas denote regions where the regression coefficients are not significantly different from zero. Dark grey areas indicate regions without observed data. PPM, posterior papillary muscle. Example interpretation: A PPM strain value of −10% (i.e. 10 units below the mean, indicating stronger contraction) is associated with a strong significant positive effect on untwisting (intense red colour) at ∼0.5 of the untwist phase.

For the PPM, a positive association with LV mid-diastolic untwisting was detected only at higher contraction magnitudes, defined as strain values more negative than the mean. In this range, increased PPM shortening was significantly correlated with pronounced untwisting during mid-diastole, whereas no significant association was observed at lower contraction magnitudes. (*Figure 4*).

A sensitivity analysis excluding participants with hypertension or diabetes was performed to assess robustness (*n* = 61 remaining) and yielded results consistent with the primary analysis (see Supplementary data online, *Figures S1* and *S2*).

## Discussion

In this study, we demonstrate that LV untwisting begins while PM contraction is still ongoing and that PM mechanics are significantly associated with the untwisting process from proto to mid-diastole.

### PM mechanics

Detailed characterization of PM mechanics in the human heart remains limited. Nonetheless, the few available reports assessing PM free strain are consistent with our findings.^13,14^ Most data on PM behaviour originate from animal studies conducted in the 1950s, predominantly in canine and equine models.^9–11,24^ These investigations consistently demonstrated that PM shortening begins during late ejection, continues through isovolumic relaxation, and frequently extends into mid-diastole. This systolic–diastolic overlap closely parallels our observations and suggests that, beyond their established role in preventing mitral valve prolapse during systole, sustained PM contraction into diastole may contribute to the LV untwisting process.

### PM LV untwist coupling

It is well established that the twisting motion of the left ventricle is primarily attributed to the helicoidal arrangement of cardiac myofibres.^1–3,15^ In contrast, the mechanisms governing LV untwisting are less clearly defined, in part because no discrete anatomical structure can be identified as its sole determinant. Prior work has proposed that elastic energy stored within the myocardial matrix during systolic deformation is released during relaxation, thereby contributing to untwisting.^6^ Although the elastic properties of myocardial proteins are undoubtedly involved, passive recoil alone is insufficient to explain the rapidity, adaptability, and fine temporal modulation of untwisting required for precise regulation of intraventricular pressure during diastole. These considerations strongly suggest that active, energy-dependent mechanisms must contribute to the fine tuning of LV untwisting, complementing passive elastic forces to meet dynamic diastolic demands.^7,25–27^

APM and PPM are positioned on opposite sides of the LV rotational axis, near the apical region. During systole, the LV undergoes a pronounced counterclockwise apical rotation driven by the helicoidal myocardial fibre architecture. As peak torsion is reached, the rotational contribution of these fibres diminishes. At this stage, as demonstrated by our findings, PM contraction persists and has not yet reached its maximal shortening. Under these conditions of declining global rotational forces, the sustained tension generated by the PMs may exert a counter-rotational effect on the apical region consistent with the significant associations between PM mechanics and LV untwisting observed in our study. Through their bilateral and apically anchored configuration, continued PM shortening, particularly of greater magnitude, as demonstrated in our results, may pull the rotated myocardium back towards its resting orientation, thereby relieving the torsional state and contributing to LV untwisting during the early and mid-untwist phase (see Supplementary data online, *Video S1*). This mechanistic view addresses important gaps in our understanding of LV untwisting. The observed temporal association of PM contraction with untwisting is consistent with a possible contribution to the transition from twist to untwist.

### Implications for disease states

The mechanistic model proposed here generates concrete, testable predictions for the disease populations not represented in the present cohort:

1. In heart failure with preserved ejection fratcion (HFpEF), in which early diastolic untwisting and untwist rate are characteristically impaired,^28^ the model predicts a reduced or temporally dissociated APM and PPM contribution to proto and mid untwist.
2. In functional mitral regurgitation, in which PM dyssynchrony has been documented,^13^ the model predicts disrupted sequential APM/PPM coupling and a measurable reduction of the mid-diastolic PM association.
3. In ischaemic heart disease, segmental PM dysfunction in the LAD (APM) or RCA/LCx (PPM) territory would be expected to disrupt the corresponding regional component of the temporal coupling. Studies in these populations, ideally with a multimodal echocardiographic/cardiovascular magnetic resonance (CMR) design, are needed to test these predictions.

### Limitations

#### Unexplained mechanisms

Our study does not explain why the PMs contract during diastole, unlike the free LV walls. These questions remain unanswered even in the previous animal studies and represent important avenues for future research.

#### Concurrent model

In this study, we only considered the concurrent effects of APM and PPM contraction on LV untwisting. However, it is plausible that time-dependent delays, cumulative contributions, and interactions between the two PMs also influence the untwisting process. Future studies employing more sophisticated approaches such as modelling the historical and cumulative effects of PM contraction may yield deeper insights into the mechanisms linking PM mechanics to LV untwisting.

#### Reproducibility

The feasibility, reproducibility, and repeatability of twist mechanics and PM strain curve measurements may raise concerns. To our knowledge, there is currently no established method to quantify inter- and intra-observer variability for curve-based measurements. To mitigate this limitation, all analyses were performed by an experienced operator, and curves were averaged over two to three cardiac cycles, which we believe substantially reduces measurement bias. We did not perform a formal inter- or intra-observer reproducibility sub-study, this is a key limitation, and a dedicated sub-study (inter-observer intraclass correlation coefficient (ICC) for peak strain magnitude and timing, plus functional ICC for the strain curve itself) is a prerequisite for clinical translation.

#### Validation and multimodal comparison

Our findings have not been replicated in an independent cohort yet, and we did not perform cross-modality validation against CMR tagging or feature tracking. There is at present no standardized CMR-based methodology for PM longitudinal strain, and cross-modal validation of PM strain is itself an open methodological question.

#### Population

We acknowledge that the inclusion of participants with hypertension represents a limitation of the present study. However, as the study was conducted in a tertiary care centre, access to truly normotensive individuals without comorbidities was limited. Consequently, the study population primarily consisted of volunteers such as medical fellows, students, and manuscript authors. Importantly, all participants with hypertension had well-controlled blood pressure and underwent comprehensive echocardiographic evaluation confirming normal cardiac structure and function. These measures were intended to minimize the potential confounding impact of hypertension on the observed findings. Nevertheless, subclinical diastolic dysfunction is common in treated hypertensive individuals despite apparently normal echocardiograms. To address this possibility, a sensitivity analysis excluding participants with hypertension or diabetes was performed and yielded results that were consistent with those obtained when hypertensive and diabetic participants were included, supporting the robustness of the findings.

## Conclusion

This study provides novel evidence that PMs actively contract during diastole in humans with echocardiographic normal hearts, temporally coinciding with and potentially facilitating LV untwisting. The significant association between PM strain and untwisting parameters suggests a potential temporal relationship of PM activity with the untwisting process. These findings challenge the traditional view of PMs as purely systolic stabilizers and instead support a dynamic, time-dependent association with diastolic ventricular mechanics. From a clinical perspective, altered PM mechanics may represent a previously unrecognized factor associated with diastolic dysfunction. Future studies are warranted to elucidate the physiological triggers of diastolic PM contraction and to determine whether these mechanisms are preserved or disrupted in pathological states.

## Supplementary Material

qyag117_Supplementary_Data

## Data Availability

Data and R code are available at https://github.com/fabian-s/PPM-Torsion.

## Lead author biography

I am a cardiologist at the Cardiology Department of Fatouma Bourguiba University Hospital, Monastir, Tunisia. My clinical and research interests focus on advanced echocardiographic imaging, particularly speckle-tracking strain analysis, to improve the understanding of myocardial mechanics and cardiovascular physiology. My work investigates myocardial deformation, left ventricular and atrial function, preload modulation, and fluid–heart interactions using quantitative imaging techniques. I have authored peer-reviewed studies on deformation imaging and strive to translate innovative imaging methodologies into clinically relevant tools for the assessment of cardiac function.
