# Supplementary material for: Temporal coupling between papillary muscle strain and left ventricular untwisting during diastole
Source: Eur Heart J Imaging Methods Pract. 2026 Jun 24;4(3):qyag117. doi: 10.1093/ehjimp/qyag117 (PMC13361982; doi:10.1093/ehjimp/qyag117)
Supplement: qyag117_Supplementary_Data [file qyag117_supplementary_data.zip › supplementary.docx]

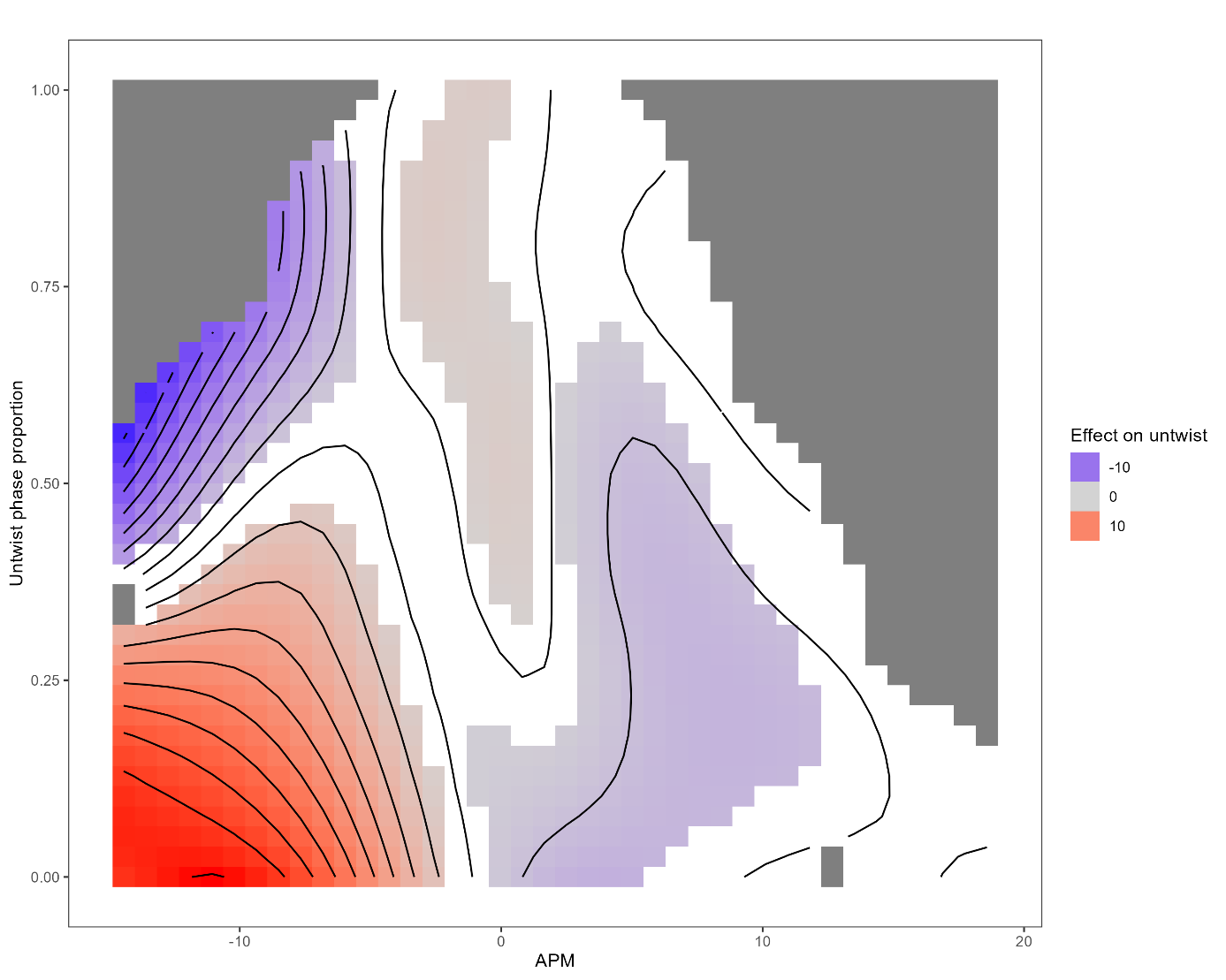


Figure 1 : **Effect of anterior papillary muscle (APM) strain on left ventricular untwisting after exclusion of participants with hypertension and diabetes (sensitivity analysis).** The y-axis represents the proportion of the untwist phase, and the x-axis indicates deviation from the mean APM strain value (%). Positive values denote above-average strain, whereas negative values indicate below-average strain (i.e., greater contraction). Color gradients reflect the direction and magnitude of the association with left ventricular untwisting; red indicates a positive association and blue a negative association. White regions denote areas where regression coefficients are not significantly different from zero, while dark gray regions indicate absence of observed data


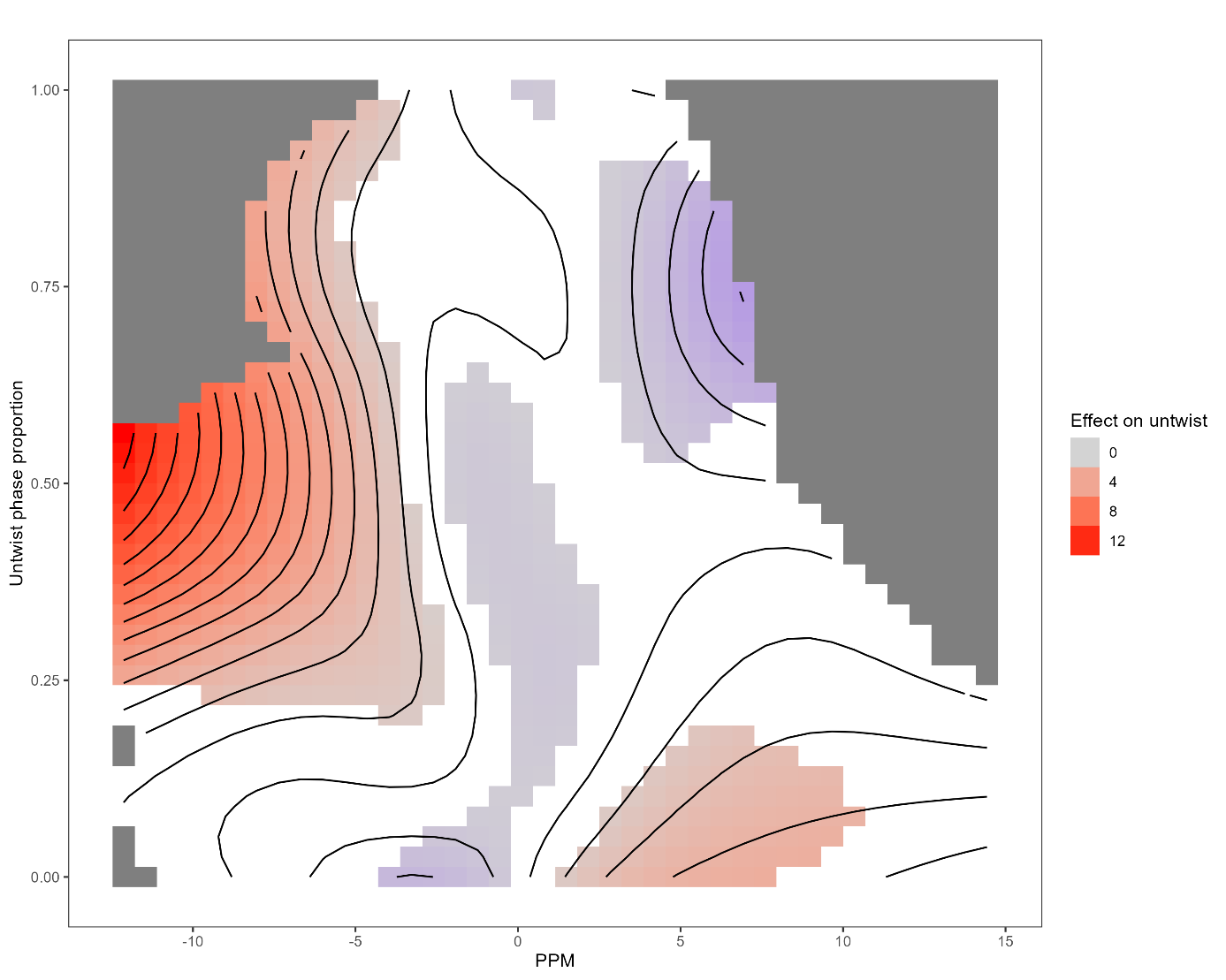


Figure 2 : **Effect of postterior papillary muscle (PPM) strain on left ventricular untwisting after exclusion of participants with hypertension and diabetes (sensitivity analysis).** The y-axis represents the proportion of the untwist phase, and the x-axis indicates deviation from the mean PPM strain value (%). Positive values denote above-average strain, whereas negative values indicate below-average strain (i.e., greater contraction). Color gradients reflect the direction and magnitude of the association with left ventricular untwisting; red indicates a positive association and blue a negative association. White regions denote areas where regression coefficients are not significantly different from zero, while dark gray regions indicate absence of observed data
